# Current concepts and controversies in the pathogenesis of Parkinson’s disease dementia and Dementia with Lewy Bodies

**DOI:** 10.12688/f1000research.11725.1

**Published:** 2017-08-30

**Authors:** Rimona S. Weil, Tammaryn L. Lashley, Jose Bras, Anette E. Schrag, Jonathan M. Schott

**Affiliations:** 1 Dementia Research Centre, UCL Institute of Neurology, London, UK; 2 Department of Molecular Neuroscience, UCL Institute of Neurology, London, UK; 3 Queen Square Brain Bank for Neurological diseases, UCL Institute of Neurology, London, UK; 4 Department of Clinical Neurosciences, UCL Institute of Neurology, London, UK

**Keywords:** Parkinson's disease dimentia, PDD, Dmentia with Lewy Bodies, DLB, neurodegeneration

## Abstract

Parkinson’s disease dementia (PDD) and dementia with Lewy bodies (DLB) are relentlessly progressive neurodegenerative disorders that are likely to represent two ends of a disease spectrum. It is well established that both are characterised pathologically by widespread cortical Lewy body deposition. However, until recently, the pathophysiological mechanisms leading to neuronal damage were not known. It was also not understood why some cells are particularly vulnerable in PDD/DLB, nor why some individuals show more aggressive and rapid dementia than others. Recent studies using animal and cell models as well as human post-mortem analyses have provided important insights into these questions. Here, we review recent developments in the pathophysiology in PDD/DLB. Specifically, we examine the role of pathological proteins other than α-synuclein, consider particular morphological and physiological features that confer vulnerabilities on some neurons rather than others, and finally examine genetic factors that may explain some of the heterogeneity between individuals with PDD/DLB.

## Introduction

Parkinson’s disease (PD) is one of the most common neurodegenerative diseases, characterised clinically by bradykinesia, rigidity and tremor. Pathologically, the cardinal features are of degeneration of dopaminergic cells in the nigrostriatal system, and by widespread intracytoplasmic Lewy bodies (LBs) and Lewy neurites (LNs) ^1^, the main component of which is α-synuclein ^2^. How Lewy pathology relates to dopaminergic degeneration and later to more widespread cell death has remained contentious ^3, 4^.

Whilst for many years PD was considered a movement disorder, in recent years a wide range of non-motor features, including cognitive impairment, have increasingly been recognised. Although subtle cognitive impairment is relatively common even at the point of diagnosis, after ten years of symptoms the population prevalence of frank dementia (so-called Parkinson’s disease dementia, or PDD) may be as high as 70% ^5^. Conversely, patients with dementia with Lewy bodies (DLB) have cognitive impairment that precedes or coincides with the development of parkinsonian signs by a (arbitrary) year or more ^6^. While the precise pathophysiological mechanisms that lead to PDD and DLB are not fully understood ^7^, neuropathologically they are difficult to differentiate ^8– 10^, apart from a higher prevalence of Alzheimer’s-like pathology in DLB than PDD ^11, 12^. In many ways, PDD and DLB are widely considered to be either end of a spectrum of one disease ^9, 13^. Considering the two diseases together as a joint entity of PDD/DLB provides a means of determining common mechanisms leading to the same end-point, and any differences potentially provide additional insights into phenotypic diversity ( Table 1).

**Table 1.** Summary of diagnostic criteria for Parkinson’s disease dementia and dementia with Lewy bodies.

**Probable Parkinson’s disease dementia (PDD) ^a^**		
Core features	Diagnosis of Parkinson’s disease (PD)	
	Progressive cognitive decline in context of established PD	Impairments in more than one domain Interferes with daily life
Associated features	Cognitive features	Attention/visuo-spatial/memory/language
	Behavioural features	Hallucinations
		Delusions
		Daytime sleepiness
		Apathy
		Mood and personality change
Absence of other abnormalities causing cognitive impairment		
**Probable dementia with Lewy bodies (DLB) ^b^**		
Essential features	Progressive cognitive decline	May have prominent attention/visuo-spatial/frontal-subcortical deficits Memory involvement more prominent in later stages Interferes with daily life
Core features (two required for diagnosis of probable DLB)	Fluctuating cognition	May have prominent attention/visuo-spatial/frontal-subcortical deficits Memory involvement more prominent in later stages
	REM sleep behaviour disorder Recurrent visual hallucinations Parkinsonism	
Supportive clinical features	Neuroleptic sensitivity Falls/syncope/loss of consciousness Severe autonomic dysfunction Hyposmia Hallucinations in other modalities Delusions Depression/apathy/anxiety	
Supportive biomarkers	Relatively preserved medial temporal lobe (magnetic resonance imaging/computed tomography) Low dopamine transporter uptake on single-photon emission computed tomography or positron emission tomography (PET) imaging Reduced occipital uptake on fludeoxyglucose-PET Prominent slow-wave on electroencephalogram	
DLB less likely	In the presence of other illnesses that could account for the clinical picture If Parkinsonian features are the only core feature or appear for the first time at a late stage of the dementia	
Temporal sequence	Dementia occurs concurrently or before onset of parkinsonism. (In research studies, the existing 1-year rule between onset of dementia and parkinsonism is still recommended.)	

^a^Adapted from 129; ^b^adapted from 6.

Although pathological markers of PDD/DLB are now well established, several key areas have until recently remained relatively less well explored. These include the pathophysiological processes that underpin PDD/DLB; mechanisms of cellular toxicity; and how the disease progresses within the brains of people affected by PDD/DLB. In addition, robust models to explain why specific brain regions or even certain neurons are more prone to involvement in PDD/DLB have been lacking, as has an understanding of the mechanisms underlying heterogeneity between individuals with PDD/DLB, including the timing of onset of cognitive decline.

Here, we review recent advances that offer new insights into these questions and shed light on the pathogenesis of PDD/DLB under five headings: (1) We review evidence that other macromolecular structures, and not just LBs, have a role in the neurodegeneration of PDD/DLB; (2) we consider recent challenges to the primacy of LBs themselves in causing cell death and dementia; (3) we consider theories for progression of pathology within individuals with PDD/DLB; (4) we examine aspects of cellular morphology and physiology that confer vulnerability to Parkinson’s pathology; and (5) we consider the role of genetic factors in the pathogenesis of sporadic PDD/DLB and what insights these provide in furthering our understanding of these diseases.

## Not just Lewy bodies

**Figure 1.**
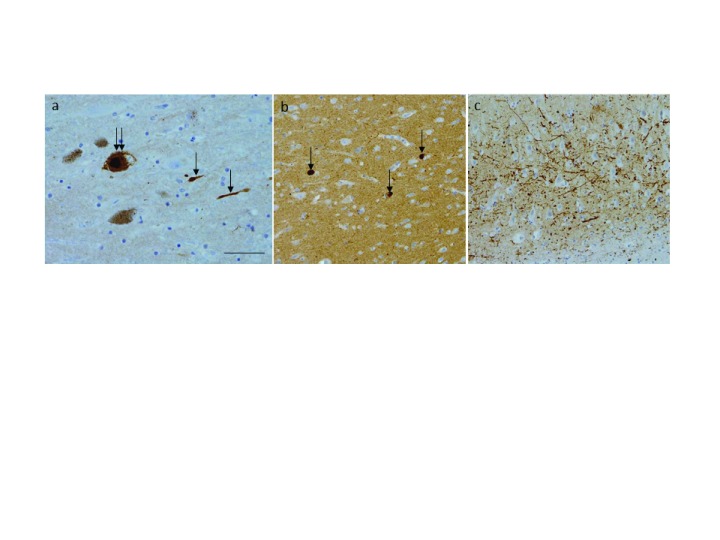
Alpha-synuclein pathology. ( **a**) Lewy body found in the dopaminergic cells of the substantia nigra (double arrow) along with Lewy neurites (arrows). ( **b**) Lewy bodies observed in the cingulate gyrus (arrows). ( **c**) A dense network of Lewy neurites in the CA2 subregion of the hippocampus. Bar = 50 µm ( **a**) and 100 µm ( **b**, **c**).

The neuropathological signatures of PD, PDD and DLB were first described by Friedrich Heinrich Lewy ^1^. He observed large eosinophilic spherical or kidney-shaped inclusions in neuronal cell bodies that were later termed Lewy bodies (LBs) ( Figure 1). He also described structures that stained less easily with acidophilic dyes and varied in morphology between short and thick or long and thread-like which were subsequently termed Lewy neurites (LNs). Immunohistochemical techniques that stained for ubiquitin ^14^ and later the use of anti-α-synuclein antibodies ^2^ revealed the extent of α-synuclein deposition in the LBs and LNs in PDD/DLB. Lewy described their occurrence in patients with paralysis agitans in various brain structures, including, but not primarily, in the substantia nigra (SN), with a diffuse and cortical distribution ^15^. Little clinical detail was given in his first descriptions, and only later was the association with dementia fully recognised and described as DLB or “diffuse Lewy body disease” ^16, 17^.

Since then, evidence has been accumulating that other pathologies are also present in patients with PD and dementia and that it is the combination of pathologies that is important for determining cognitive impairment in the face of Parkinson’s pathology. Thus, the combination of Lewy pathology and Alzheimer’s disease (AD) pathology (fibrillary β-amyloid and intraneuronal tangles consisting of hyperhosphorylated tau) predicts dementia in PD better than the severity of any single pathology ^18^; and in clinical studies in patients with newly diagnosed PD, cerebrospinal fluid (CSF) biomarker evidence for β-amyloid pathology (for example, low concentrations of Aβ1-42) is a significant predictor of subsequent cognitive impairment ^19^.

Large-scale studies comparing patterns of CSF biomarkers between patients with DLB and PDD show that lower levels of Aβ1-42 (combined with higher tau levels) are associated with DLB rather than PDD and are seen particularly in patients with more rapid dementia ^20– 22^. CSF Aβ1-42 is inversely related to brain density of amyloid plaques ^23^ and therefore lower CSF Aβ1-42 concentration is supportive of higher brain amyloid in these patients. Similarly, in positron emission tomography imaging studies using amyloid radioligands, higher levels of amyloid are seen in patients with DLB than PDD and in PD patients with more rapid cognitive progression ^24– 26^. Patients with the combination of Lewy-related pathology and evidence for brain β-amyloid accumulation have a more aggressive disease course and have shorter survival times and higher incidence of cognitive dysfunction even compared with pure AD ^27– 31^, and these proteins may mutually promote each other’s aggregation ^32^. Thus, in a large retrospective study, Irwin and colleagues ^8^ showed a strong correlation between the extent of neurofibrillary tangles, neuritic plaques and α-synuclein, suggesting synergistic effects of AD and α-synuclein pathology. Patients with PDD/DLB with more cortical β-amyloid plaques also have more cortical α-synuclein aggregates ^33, 34^. Conversely, even in “pure” young-onset familial (autosomal dominant) AD, a high proportion of patients have LB pathology at autopsy (although in these cases it tends to localise to the amygdala) ^35, 36^ and α-synuclein accumulates within amyloid plaques of dystrophic neurites ^37^. This synergy is also seen in mouse models of PDD/DLB: transgenic mice overexpressing both human β-amyloid and α-synuclein have higher levels of LB pathology and greater memory deficits than mice expressing just α-synuclein ^38^; and double transgenic mice overexpressing two AD-related genes (amyloid precursor protein and presenilin 1) have greater amounts of α-synuclein as well as β-amyloid than mice transgenic for one mutation alone ^39^.

A potential mechanism for this synergy is through phosphorylation. α-synuclein can induce tau hyperphosphorylation ^40, 41^, thereby promoting neurofibrillary tau tangle formation ^41, 42^. Similarly, in PDD/DLB, the main modification of α-synuclein is Ser129 phosphorylation ^43^. An estimated 90% of α-synuclein in LBs and LNs is phosphorylated at Ser129, compared with only 4% in unaffected brains ^44^. This changes its solubility and enhances the tendency for α-synuclein aggregation. Higher levels of phosphorylated α-synuclein are seen at earlier stages of PDD/DLB than LB pathology ^45^, and phosphorylated α-synuclein levels correlate with disease severity ^46, 47^. Recently, Swirski and colleagues ^48^ shed light on the synergistic relationship between β-amyloid and α-synuclein, demonstrating correlations between phosphorylated Ser129 α-synuclein and the amount of β-amyloid and between the proportion of α-synuclein phosphorylated at Ser129 and antemortem cognitive function. Importantly, by taking cells that overexpressed α-synuclein, exposing them to toxic forms of β-amyloid (Aβ1-42) and showing that this led to a higher proportion of α-synuclein phosphorylated at Ser129, they were able to show a causative role for β-amyloid in pathological synuclein phosphorylation. Taken together, these studies show that the synergistic relationship between AD pathology and α-synuclein is bidirectional and that each protein synergises the other.

## Not even Lewy bodies

Although Lewy bodies are considered the neuropathological signature of PD, the severity of clinical symptoms, disease duration, and presence of cognitive decline or visual hallucinations (core features of PDD/DLB) do not correlate with LB density ^3, 49– 51^; and in some patients with PDD, no LBs are seen in cortical regions or even outside the brainstem ^52^. A recent neuropathological study showed that cell loss can precede LB accumulation ^53^, calling into question the hypothesis that LBs are the toxic agents in PD that drive neurodegeneration ^54^. One proposed explanation is that it is the precursors of LBs (which cannot be seen by light microscope), rather than the LBs themselves, that precipitate neurodegeneration ^55^. Although there is no correlation between LB and nigral neuronal loss, there is a correlation between neuronal loss and total α-synuclein aggregate burden ^56, 57^; and evidence suggests that initial amorphous α-synuclein deposits known as pale bodies and pale neurites may mediate cell damage and later evolve into more aggregated LBs ^55^.

A related theory proposes that neurodegeneration and cell death are not caused by LBs but that instead LBs provide protection from α-synuclein aggregates ^58, 59^. In this way, LBs and LNs may be an indirect indicator of disease but do not reflect the full extent of neurodegenerative pathology ^60^, similar to that proposed for β-amyloid plaques in AD ^61, 62^.

The recent discovery of different strains of α-synuclein is an intriguing potential explanation for the heterogeneity of synucleiopathies between individuals ^63^. Bousset and colleagues generated two distinct high-molecular-weight assemblies of α-synuclein: one with a cylindrical and fibrillar structure and one with a more flat and ribbon-like structure, as shown by transmission electron microscopy and x-ray diffraction ^63^. Crucially, the fibrillar conformation was more toxic to cells, inducing caspase-3 activation and reactive oxygen species and showing a higher propensity to bind and penetrate cells. Moreover, the two strains showed differential abilities to induce tau aggregation both in cultured neurons and *in vivo* ^64^ (and see review ^65^). Thus, and again in line with proposed models for AD, pathological processes upstream of the Lewy bodies may be more influential in disease progression, and more aggressive disease may relate to distinct strains of α-synuclein.

## Not “prion-like” spread?

Braak and colleagues proposed that Lewy pathology could spread from cell to cell in a manner similar to the proposed spread of prion proteins. This followed detailed observations that patients who had died at earlier clinical stages of PD had Lewy pathology confined to the lower brainstem but that those patients who succumbed at later stages of disease had more abundant Lewy pathology in the upper brainstem and then cortex ^66, 67^. The authors hypothesised that the disease started in the brainstem before spreading into the striatum and then the cortex ^68^; and more recent work has suggested disease originating in the gut and nose ^69– 71^. This model gathered support when foetal tissue grafts transplanted into human striatum were found to show Lewy-like inclusions within decades of transplantation ^72^. A series of investigations followed, aimed at showing transmission of α-synuclein between cells. In cellular models, transfection of α-synuclein precursors into cells expressing α-synuclein induced Lewy body–like inclusions ^73, 74^. In mice, synthetic α-synuclein fibrils were shown to spread from the site of injection to synaptically connected structures, creating Lewy-like pathology ^75, 76^. Similar observations were made when young mice were injected in various brain regions with extracts from the brains of older motor-impaired mice ^77^.

However, these and other studies do not yet provide incontrovertible evidence that α-synuclein induces pathology or even spreads between cells. Not all cases find Lewy-like inclusions in transplanted tissue ^78^ and some find them in only a minority of grafted cells ^79^. The concentrations of α-synuclein precursors in cellular models are typically above physiological levels of α-synuclein ^80^; and in animal models, factors other than α-synuclein itself could be promoting α-synuclein aggregation, including damage from injection sites or local inflammation ^80^. Indeed, more detailed analysis of one animal model revealed that the α-synuclein inclusions appeared randomly without any evidence of spread between cells ^81^.

For a mechanism involving trans-synaptic spread, it would be expected that cells with the greatest synaptic connectivity to regions affected in early PD would be at greatest risk of developing Lewy pathology. However, this is not the case; mouse studies show that regions with strong connectivity to the SN do not show the most abundant Lewy pathology ^82^ (see also 83).

Instead, theories are emerging to suggest that Braak and colleagues’ observations of a stereotypical pattern of involvement of particular brain regions with disease severity (inevitably based on extrapolation from cross-sectional pathological observations) reflect *selective vulnerabilities* of specific cells that tend to be affected first in PD ^80, 83, 84^, rather than the physical transmission of a pathological agent. It is also possible that the pattern of “spread” reflects a combination of processes: with cell-to-cell propagation starting in response to injury or inflammation, at particular sites and affecting particular neuronal populations, before spreading through networks of similar cells that are similarly vulnerable ^85^.

Indeed, it is these selective vulnerabilities and the pattern of involvement that may reflect the heterogeneity across the spectrum of PDD/DLB. Many of the cells affected in PD are key neurons in the neuromodulatory control networks. These share several notable traits: they are all characterised by long and highly branched axons and a large number of transmitter release sites. New models propose that it is these common features that predispose particular neurons to be affected early in PD: we next examine the evidence for each of these in turn.

## Cell vulnerabilities

### Long axons

The importance of axons and striatal terminals in the pathogenesis of PD has been known for some time. Loss of dopamine is more profound at the axon terminals in the caudate and putamen than is loss of nigral neurons ^86– 89^ (reviewed in 90), suggesting that degeneration is greatest in distal parts of the cell. Early studies showed that vesicles accumulate along cell processes and axons and close to α-synuclein inclusions in Parkinson’s ^91, 92^. Others demonstrated the presence of pathological α-synuclein aggregates in axon terminals of the hippocampus in the brains of people with PD ^93^. Together, these studies suggest that blocked axons or impairment of traffic within axons may be important in PD.

Orimo and colleagues examined cardiac sympathetic axons to study the chronological sequence of events at the cellular level in PD. They showed that α-synuclein aggregates accumulate in the distal axons of the cardiac sympathetic nerves and that this axonal accumulation occurs prior to degeneration of the cardiac sympathetic nerve itself ^94, 95^.

More recent neuro-histological studies support the theory that axonal involvement is a critical, early feature in PD and that α-synuclein aggregation starts in the axonal compartment and progresses back towards the cell body. Chung and colleagues ^96^ demonstrated that α-synuclein overexpression in rats leads to axons becoming dystrophic, with alterations in axonal transport. Importantly, all of these changes preceded neuronal loss. Another study showed that, at the point of diagnosis, there is a far higher number of nigral neurons with axon degeneration than neuronal loss ^90^.

This central role of the axon in PD pathogenesis is supported by studies of transgenic mice carrying mutations in human *LRRK2* ^97^, one of the most common genetic causes of familial PD ^98^. These mice show pathology in dopaminergic axons but no loss of dopaminergic neurons themselves. Moreover, at the level of the single axon, staining for tyroxine hydroxylase reveals fragmentation of the axons as well as axonal spheroids and dystrophic neurites.

A recent detailed study examining critical axonal transport motor proteins ^99^ found that levels of a major microtubule-based motor protein, known as conventional kinesin, are depleted in nigral neurons in PD at the earliest stages of disease. These reductions preceded changes in dopaminergic phenotypic markers and were more pronounced in the presence of α-synuclein. Similar findings of reduced axonal transport motor proteins were observed in a rat model of PD with overexpression of α-synuclein. One potential mechanism is that α-synuclein inclusions do not simply occlude the axon, but damage the cell by affecting specific aspects of organelle transport ^100, 101^. This may have greater impact in cells with particularly long axons.

The importance of long thin axons in conferring vulnerability to Lewy-related pathology is supported by the observation that other neurones that are preferentially affected in PDD/DLB also display this anatomical configuration. For example, cholinergic cells of the nucleus basalis of Meynert are strongly implicated in the pathogenesis of dementia in PD ^102, 103^. In mice, these show extreme length and a complex branching structure, and it is estimated that in humans, these cells may have an average length of approximately 100 metres ^104^. Serotonergic cells of the raphe nucleus also show extensive axon projections ^105^, and the long unmyelinated axons of the peripheral autonomic nervous system may provide an explanation for the early and prominent involvement of autonomic symptoms in both DLB and PDD. Thus, the anatomical configuration of neurons may reflect their specific vulnerability to PD progression and also to the development of dementia in patients with PD pathology.

### Hyperbranching axons

A further morphological feature common to dopaminergic, noradrenergic, cholinergic and serotonergic systems is the hyperbranching of long axons that project widely to innervate multiple brain regions ^106^. In a recent study, Kanazawa and colleagues ^55^ examined how α-synuclein is aggregated and extends into neurites. They used a combination of fluorescent and haematoxylin-and-eosin staining and both light and electron microscopy that allowed them to quantify LNs. They found that LNs are frequently clustered around branch points, at the point where a collateral leaves the main axon, and that the α-synuclein then spreads contiguously along the neuronal axon. This finding that axonal α-synuclein deposition is preferentially seen around branching points could explain why axons that are highly divergent are more susceptible to α-synuclein deposition and why axons distal to the branching will be more vulnerable ^55^. This is indeed the case. Axons originating in the ventrolateral part of the SN, one of the most vulnerable systems in PD, are highly collateralised and branching, on single-cell labelling studies ^107, 108^, and cholinergic and serotonergic neurons show similarly long and hyperbranching configurations ^104, 105^.

### Synapses

The synapse is another potential location for early involvement in PD. α-synuclein in its physiological form localises to the presynaptic terminal, and evidence is emerging that loss of synaptic connectivity may produce neurodegeneration prior to nerve cell loss. Kramer and colleagues ^109^ used a technique involving paraffin-embedded tissue blotting that can more sensitively detect the topographic location of protein aggregates. In this way, they localised dense aggregates of α-synuclein in the synapses throughout the cortex of patients with DLB. A similar pattern of α-synuclein deposition was found in patients with PD ^110^. A far higher proportion of α-synuclein localises to presynaptic terminals as aggregates than can be found within LBs when measured using a highly sensitive protein aggregate filtration assay ^109, 111^. This presynaptic predilection of α-synuclein has been replicated in mouse models. Presynaptic microaggregates of α-synuclein have been detected in mice overexpressing different forms of α-synuclein ^112– 114^, often in the absence of cell death, suggesting that presynaptic involvement is an event that precedes neuronal death. These presynaptic α-synuclein microaggregates can then impact on post-synaptic dendritic spines. When the dendritic tree of individual cells is visualised by using a silver impregnation technique, almost complete loss of the dendritic spines in frontal cortical neurons can be found in patients with DLB compared with age-matched controls ^109^. Similar loss of dendritic spines is seen in the striatum and SN in PD ^115, 116^. The precise pathophysiological cascade is not yet clear but may involve aggregation of α-synuclein starting at either the synapse or axon branching points that then influence vesicle trafficking and impair neurotransmitter release. This causes post-synaptic dendritic spines to degenerate, with loss of synaptic connections ^60^.

### Common physiological phenotype

Instead of considering only cellular morphology, a recent influential model proposes that it is the shared physiology of vulnerable cells ^83, 106^ that is critical in the pathogenesis of PD. Neurons that are most vulnerable in PD (SN, locus coeruleus, and pedunculopontine nucleus) all share the propensity to autonomous spiking, in the absence of synaptic input. They all show large fluctuations in calcium concentrations and low intrinsic calcium buffering ^117^. Together, these traits generate heavy metabolic demands on the cells that are particularly carried by the mitochondria ^83^. As mitochondrial and proteosomal function degrades with age, these neurons are less able to handle the burden of α-synuclein aggregation. This may explain why mutations affecting mitochondrial function are highly represented in PD ^118, 119^. Which of these vulnerabilities—long hyperbranching axons, synapses, or autonomous cell spiking—is most critical in the pathophysiology of PD will need to be specifically tested. Ultimately, it is likely that some or all of these features combine to make these cells more vulnerable in PD. It is also possible that some degree of spreading of α-synuclein does occur but is limited to cells that show particular vulnerability to handling excessive misfolded α-synuclein. Alternatively, the apparent spread of disease instead may reflect the relative propensity of α-synuclein to form in these structures.

## Genetics

The genetic underpinnings of PDD/DLB have been vastly understudied. Two main reasons may have contributed to this: first, the fact that large, homogeneous cohorts of PDD/DLB cases have been difficult to assemble and thus large-scale genetic studies have not been possible; second, because we do not tend to see familial aggregation of DLB, as we do in some instances in PD or AD, the general reasoning has been that there is no genetic basis for this disorder. However, the last few years have started to shift this notion, much like the early ’90s did for PD and the early ’70s for AD.

One of the first hints that genetics played a role in DLB came from the study of *GBA*. Homozygous mutations in *GBA* are known to cause a lysosomal storage disorder (Gaucher disease), whereas the same mutations when heterozygous increase risk for PD ^120^. In DLB, a similar effect of these mutations was identified ^121^. The immediate suggestion from these data is that there is an identical underlying lysosomal dysfunction occurring in both diseases. More recently, an association study showed that common variability is also involved in DLB. Variants at the *APOE* and *SNCA* loci were shown to be associated with DLB in a cohort of approximately 800 cases ^122^. Interestingly, whereas the *APOE* association was identical to the one seen in AD, the association at *SNCA* was completely independent of the one seen in PD. These results suggest that, although the same locus is involved in both diseases, the way in which this involvement occurs is different. It is tempting to speculate that perhaps the differential association underlies differential gene expression of *SNCA*, potentially in a tissue-specific manner.

It was also shown that, in addition to sharing two loci of association with PD and AD, there is a substantial amount of genetic correlation between DLB and both PD and AD ^123^ and that the heritability of DLB can be estimated to be approximately 30%, similar to estimates for PD ^124^.

Taken together, these data strongly suggest not only that DLB has a genetic component but also that this component has a unique architecture when compared with PD and AD leading to the specific phenotype of DLB. The genetic differences between PDD and DLB have, so far, not been studied in detail. In PDD, factors that predispose to earlier dementia may have some genetic underpinnings. For example, REM sleep behaviour disorder, which is predictive of cognitive involvement when it occurs in patients with PD ^19^, is more common in patients carrying *GBA* ^125^ and *SNCA* mutations ^126, 127^ and less common in patients carrying *LRRK2* mutations ^128^.

However, the recent genetic data described above suggest that categorising diseases in a binary fashion might not be adequate and that it is the varying polygenic characteristics between individuals that underpin heterogeneity in patients with PDD/DLB.

As our understanding of these factors increases, these concepts will increasingly be included in our thinking of disease pathobiology; and as we move to an era of personalised medicine, genetic risk is likely to be used diagnostically and to predict which patients with PD pathology are at risk of developing cognitive impairment, perhaps replacing the arbitrary clinical criteria that are currently used to distinguish DLB from PDD.

## Summary

Recent cellular, animal, post-mortem and genetic studies are beginning to shed light on key and previously unknown aspects of the pathophysiological mechanisms that underlie PDD/DLB. These include insights into which aspects of PD pathology are most closely linked to cognitive decline, the interaction between AD and PD pathology, and the cellular and morphological features that may explain disease spread and selective neuronal vulnerability. Although genetic studies of DLB/PDD are in their infancy, results to date show commonalities with both PD and AD but also notable differences. As our understanding of the basic pathophysiology of these conditions expands and as links between genotype, pathological mechanisms and phenotype are established, prospects for personalised medicine will increase: these include prediction of not only which patients will develop cognitive impairment but when. Importantly, these observations may also provide important insights into the mechanisms underlying neurodegenerative diseases more generally as well as new targets for novel disease-modifying therapy to prevent dementia in PD and DLB.

## Abbreviations

AD, Alzheimer’s disease; CSF, cerebrospinal fluid; DLB, dementia with Lewy bodies; LB, Lewy body; LN, Lewy neurite; PD, Parkinson’s disease; PDD, Parkinson’s disease dementia; SN, substantia nigra.
